# Tone of mood in new mother and attachment to her partner

**DOI:** 10.1192/j.eurpsy.2025.2146

**Published:** 2025-08-26

**Authors:** I. S. Lancia, G. M. Festa, A. Attouchi, F. De Pasquale, M. Manganozzi, M. Civino, L. M. Colone

**Affiliations:** 1 Interdisciplinary Institute of Advanced Clinical Training; 2 Pontifical Faculty of Educational Sciences «AUXILIUM; 3 Interdisciplinary Institute of Advanced Clinical Training «IACT», Rome, Italy

## Abstract

**Introduction:**

Mood in puerperium is a subject of great interest since it can undergo variations in such a significant period of a woman’s life as motherhood. It is equally true that the presence of a partner with a secure attachment style can constitute a stable base for a woman to lean and to rely, while it is more likely to hypothesize cracks in mood in absence of effective support in the relational context.

**Objectives:**

The aim of this research is to analyse the trend of puerperal mood in women in the period immediately following the delibery and after a few months. The study also examines the attachment styles of the partners of these women and the relationship between mood in women and the attachment of their partners.

**Methods:**

The study was conducted on a sample of women and their partners.

The women were administered 2 administrations of the Edinburgh Peripartum Depression Scale (EPDS): the first within 24 hours of delivery and the second four months after delivery.

The Relationship Questionnaire (RQ) was administered to the male partners immediately after the evnt of delivery.

The data were analyzed from a statistical point of view, with analysis of variance (ANOVA) and post hoc tests.

**Results:**

A 2-way ANOVA with repeated measures was performed using the different attachment styles as emerged from the RQ (secure attachment and insecure attachment) in the partner group as the independent variable and the EDPS scores in the 2 times (EPDS1, EPDS2) of the women themselves as the dependent variable. The ANOVA described the main effect of Time as significant (F (1, 14) = 5.79; p < .05). The post hoc (LSD - Least Significant Difference test) highlights how there is a significant decrease in the EPDS score of women in the before-after comparison (M = 8.71 vs M = 4.93; p = .005) considering, as an independent variable, the secure attachment group of partners. While this decrease is not significant in the group of partners with insecure attachment.

**Conclusions:**

The initial data of this study suggest that security in the attachment style of the partner can favor an increase in the mood of the woman after a delivery. In particular, the research data tend to highlight a significant improvement in the mood of women 4 months after giving birth. The proximity of a partner with secure attachment therefore seems to favor a good mood in the woman in pueperium.

**Disclosure of Interest:**

None Declared

